# Mapping Migratory Bird Prevalence Using Remote Sensing Data Fusion

**DOI:** 10.1371/journal.pone.0028922

**Published:** 2012-01-03

**Authors:** Anu Swatantran, Ralph Dubayah, Scott Goetz, Michelle Hofton, Matthew G. Betts, Mindy Sun, Marc Simard, Richard Holmes

**Affiliations:** 1 University of Maryland, College Park, Maryland, United States of America; 2 Woods Hole Research Center, Falmouth, Massachusetts, United States of America; 3 The Department of Forest Ecosystems and Society, Oregon State University, Corvallis, Oregon, United States of America; 4 Jet Propulsion Laboratory, California Institute of Technology, Pasadena, California, United States of America; 5 Dartmouth College, Hanover, New Hampshire, United States of America; University of Bristol, United Kingdom

## Abstract

**Background:**

Improved maps of species distributions are important for effective management of wildlife under increasing anthropogenic pressures. Recent advances in lidar and radar remote sensing have shown considerable potential for mapping forest structure and habitat characteristics across landscapes. However, their relative efficacies and integrated use in habitat mapping remain largely unexplored. We evaluated the use of lidar, radar and multispectral remote sensing data in predicting multi-year bird detections or prevalence for 8 migratory songbird species in the unfragmented temperate deciduous forests of New Hampshire, USA.

**Methodology and Principal Findings:**

A set of 104 predictor variables describing vegetation vertical structure and variability from lidar, phenology from multispectral data and backscatter properties from radar data were derived. We tested the accuracies of these variables in predicting prevalence using Random Forests regression models. All data sets showed more than 30% predictive power with radar models having the lowest and multi-sensor synergy (“fusion”) models having highest accuracies. Fusion explained between 54% and 75% variance in prevalence for all the birds considered. Stem density from discrete return lidar and phenology from multispectral data were among the best predictors. Further analysis revealed different relationships between the remote sensing metrics and bird prevalence. Spatial maps of prevalence were consistent with known habitat preferences for the bird species.

**Conclusion and Significance:**

Our results highlight the potential of integrating multiple remote sensing data sets using machine-learning methods to improve habitat mapping. Multi-dimensional habitat structure maps such as those generated from this study can significantly advance forest management and ecological research by facilitating fine-scale studies at both stand and landscape level.

## Introduction

Improved maps of species distributions are critical for implementing effective conservation plans under increasing habitat loss from anthropogenic perturbations [1]–[3]. While environmental and climatic variables affect wildlife habitats in several ways, vegetation structure is one of the most important factors influencing habitat use, particularly in bird species [4], [5]. Vegetation structure influences foraging behavior [6], food abundance, nesting patterns and breeding success, which contribute to long-term persistence of bird populations. Structural characteristics in forests consistently occupied by bird species may therefore be indicators of habitat quality [1], [7], [8]. Management efforts to adequately conserve high quality habitats across landscapes consequently require extensive spatial information on forest structural characteristics and floristics. With advances in remote sensing technology, newer data with complementary attributes are increasingly available [9]. The simultaneous use of these data in physical or statistical models is commonly termed “multi-sensor fusion” [10] and has emerged as a promising approach for optimizing existing remote sensing capabilities to improve forest structure and habitat mapping [9], [11].

Multispectral data have long been used to map habitat preferences by relating species occurrence/abundance to the spatial distribution of vegetation across landscapes. These vegetation characteristics have commonly included land cover [12], phenology [13], patch size, and fragmentation [12], [14] and are mostly related to vegetation class (e.g. deciduous and conifer) and their spatial attributes. However, in situ field and other studies have postulated that vertical characteristics of the forest, such as canopy height, foliar profiles, and layering, are of equal or greater importance in explaining the abundance and diversity of species [4], [5]. This vertical dimension is difficult to obtain from multispectral data such as Landsat or MODIS [15].

Light detection and ranging (Lidar) provides accurate measurements of vertical vegetation structure and is of considerable value in ecological applications [16]–[18]. Lidar instruments essentially record the time taken by a laser pulse to reach the earth's surface or canopy top from an airplane/spacecraft and return. The laser beam interacts with canopy elements and topography to produce a record of the vertical distribution of canopy surfaces from which habitat characteristics can be derived [15], [19]. Synthetic Aperture Radar (SAR, hereafter ‘radar’) instruments record backscattered radiation from the Earth's surface in the microwave region of the electromagnetic spectrum. Radar sensors, depending on the wavelength used, are sensitive to larger elements of the canopy, such as branches and boles, and can be correlated to canopy volume, basal area, and biomass between 100–150 tons/ha (50–75 tons C/ha) [20], [21]. Radar data are also effective for mapping spatial variability in landcover and detecting disturbances. Lidar provides exquisite vertical canopy characterization, but only over spatially limited areas; radar provides large-area mapping of canopy volume but in the case of SAR only scant information on height and the vertical leaf distribution. Importantly, radar data can be obtained over large areas quickly and can be used regardless of cloud cover, in contrast to current capacity lidar. Fusion of lidar with radar and multispectral data may increase our ability to map vegetation structure, which is important for habitat studies at landscape scales. However, analyses of the efficacy of metrics derived from these data, their accuracies and combined use for mapping species habitats are needed [9].

A major obstacle in assessing multi-sensor fusion for habitat studies has been the lack contemporaneous remote sensing data that are coincident with spatially explicit wildlife data. Recently an experiment in support of NASA's DESDynI (Deformation Ecosystem Structure and Dynamics of Ice) mission was conducted at the Hubbard Brook Experimental Forest (HBEF) in New Hampshire to assess multi-sensor fusion for aboveground biomass estimation. This experiment utilized a suite of new and existing remote sensing data that had been obtained over the forest including radar, lidar, and multispectral imagery. Additionally, long-term, quantitative data on bird abundances and distributions from this study site were available for comparisons. Forest bird populations, most of which are Neotropical migrants, have been monitored at the HBEF since the late 1960s. Bird community composition, guild structure [22], foraging behavior [6], [23] and long-term trends have been extensively studied for several species [24], [25]. The availability of wildlife data along with the wide range of remote sensing data provides an unparalleled opportunity to explore multi-sensor fusion for habitat mapping and this fusion is the central goal of the research presented here.

Our objective is to evaluate statistical fusion of multi-sensor data in predicting multi-year bird detections (hereafter, “prevalence”) for eight migratory songbird species in the HBEF. Forest structural attributes, such as height and cover, as well as a suite of other habitat parameters either known or hypothesized to be important for these species are derived from remote sensing data. We test predictive capabilities of the different data sets individually and in combination with each other using machine-learning methods. We further analyze the importance of these predictor variables to determine which are most useful in describing bird habitat characteristics. Finally, we map prevalence across the landscape and compare spatial patterns with known habitat preferences for each species.

## Methods

### Study Area & Bird Observations

The HBEF is a bowl-shaped watershed located in the White Mountains of New Hampshire, USA covering an area of 3,160 ha with elevations ranging from 220 m to 1,015 m [26]. Slopes are predominantly north and south facing with an average slope of 16%. Dominant deciduous tree species at lower elevations include beech (*Fagus grandiflora*) and sugar maple (*Acer saccharum*). At higher elevations, forests are dominated by birch (*Betula sp*) and conifers such as balsam fir (*Abies balsamica*) and red spruce (*Picea rubens*). Understory vegetation includes saplings of dominant trees, striped maple (*Acer pensylvanicum*), mountain maple (*Acer spicatum*), hobblebush (*Viburnum alnifolium*), many herbs, and ferns. The HBEF is a long-term ecological research (LTER) site and is representative of northern hardwood forests. Detailed site characteristics can be found in [25].

**Figure 1:**
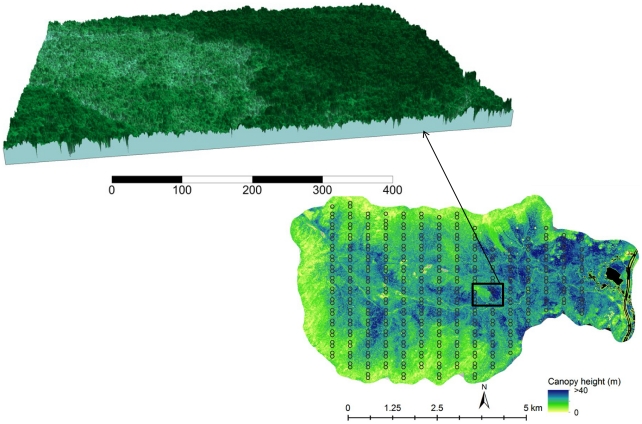
DRL canopy height map showing bird census plot locations (small circles) and canopy height model showing individual tree crowns.

Bird observation data were collected between 1999 and 2008 [7] over a grid of 371 plots laid out in north south transects across the study area (Fig. 1) [27]. Bird sightings were recorded for 10 minutes within a radius of 50 m around each plot center (0.79 ha area), two or three times every year during the peak breeding season, following point count monitoring methods [28]. Bird counts were then used to calculate multi-year detection (prevalence) over the 9-year time interval. Prevalence values ranged from 0 for no detection to 9 for detection in all years. We focused on 8 bird species (Table 1) where preliminary analyses showed strong relationships between lidar metrics and bird prevalence (more than 30% variance explained by lidar). These included the blackpoll warbler (BLPW), black-throated blue warbler (BTBW), magnolia warbler (MAWA), yellow-rumped warbler (MYWA), ovenbird (OVEN), red-eyed vireo (REVI), dark-eyed junco (DEJU), and the yellow-bellied flycatcher (YBFL). Detailed descriptions of habitat characteristics and bird data collection may be found in [22]and [29].

**Table 1:** Common and scientific names of songbird species at HBEF.

Common Name	Code	Scientific Name	General Habitat Requirements
blackpoll Warbler	BLPW	*Dendroica striata*	Coniferous forests, wide altitudinal gradient [29].
black-throated blue Warbler	BTBW	*Dendroica caerulescens*	Deciduous forests in low elevation areas with well developed understory [1], [3], [22], [29].
magnolia warbler	MAWA	*Dendroica magnolia*	Coniferous spruce-fir forests. Broad tolerance for size class, narrow tolerance for cover type [5], [29].
yellow-rumped warbler	MYWA	*Dendroica coronata*	Coniferous forests and deciduous with presence of some conifers, particularly red spruce [29].
ovenbird	OVEN	*Seiurus aurocapillus*	Low elevation deciduous forests, undisturbed mature stands, ground forager [5], [29].
red-eyed vireo	REVI	*Vireo olivaceus*	Low elevation deciduous forests [29].
dark eyed junco	DEJU	*Junco hyemalis*	Coniferous forests, higher elevation [29].
yellow-bellied flycatcher	YBFL	*Empidonax flaviventris*	Coniferous forests, higher elevation [29].

### Remote Sensing Data

#### Multispectral Data

Landsat ETM+ images acquired in August 1999 and late October 2000 were corrected for Earth-Sun distances and solar zenith angle variations, converted into top-of-atmosphere reflectance and geo-referenced [30]. The Normalized Difference Vegetation Index (NDVI) was calculated for both images. We used the NDVI as a measure of greenness and the difference between NDVI from leaf-on and leaf-off seasons as a measure of deciduousness. These variables were found to be important in determining habitat quality for the black-throated blue warbler in a previous study [1].

#### Lidar Data

Lidar data can be classified as discrete return or waveform digitizing based on the number of energy returns recorded by the sensor. Discrete return lidar instruments (DRL) record two or more returns, namely one from the ground, one from the top of the canopy and some number in between [15]. Full waveform digitizing lidar instruments record the entire outgoing and return signal to provide a waveform with amplitudes proportional to the vertical distribution of canopy material within a footprint [15]. Both DRL and waveform lidar data have been used to map canopy height, cover and aboveground biomass, in addition to sub-canopy topography [16], [31]–[34]. Many recent studies have also shown the potential of the two types of lidar in mapping habitat characteristics but few have compared their relative efficacies in such mapping efforts.

We used DRL data collected over the study area in September 2009 with at least one shot per sq. m and up to four vertical returns. First returns from lidar point cloud data were interpolated to create a digital surface model (DSM) of canopy top with a resolution of 0.5 m. Similarly, last returns were isolated and interpolated to obtain a digital elevation model (DEM) at the same resolution. A canopy height model (CHM) was derived by subtracting ground elevation from the digital surface model. The high spatial resolution of the CHM made it possible to identify dominant and co-dominant tree crowns. We used an adaptive ‘local maxima’ filtering algorithm (TreeVaW) [35] to identify trees from the CHM and obtain crown radii and height. The algorithm was calibrated using field measurements of canopy height and crown radii collected over the HBEF in 2009. Individual trees identified by the algorithm were used to calculate stem density per hectare. Crown area-weighted height for each plot was then calculated as follows:
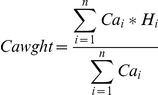

*Ca* = crown area of each tree, H = height of each tree from DRL data and *n* = number of trees in each plot. This metric is analogous to basal area weighted height (Lorey's height) measured in the field [36].

In contrast, the Laser Vegetation Imaging sensor (LVIS) is a medium-footprint (25 m diameter), full-waveform digitizing lidar developed at NASA's Goddard Space Flight Center [37]. LVIS data were acquired over New Hampshire in the summer of 2009 with trees in leaf-on condition. Canopy top was detected by finding the lidar return greater than the noise threshold at the top of the waveform. Comparisons between DRL data and LVIS waveforms showed that DRL data provided accurate measurements of ground elevation but underestimated canopy top elevations. We therefore used ground elevation from high-resolution DRL data to account for potential ground finding errors in LVIS algorithms but retained LVIS canopy top heights to calculate waveform metrics. Canopy height (RH100) was calculated by subtracting the average DRL ground elevation within each LVIS footprint from the canopy top height. Quantile energy metrics i.e. heights of 25% (RH25), 50% (RH50) and 75% (RH75) energy return were calculated from the waveform in a similar manner [38]. The correlation between LVIS canopy heights and maximum DRL height within LVIS footprints increased by 25% (R^2^ = 0.78, RMSE = 2.09 m, n = 150554 footprints) after ground correction of LVIS data. Total canopy cover was calculated from the normalized cumulative laser energy return following methods in [39]. We hypothesized that quantifying the amount of foliage at different levels within the canopy possibly could explain bird occurrence/prevalence better than other waveform metrics. We therefore calculated canopy cover at 5 m height intervals from the cumulative energy return between ground and 40 m, resulting in 8 metrics that approximated the foliage profile.

#### Radar Data

The Uninhabited Aerial Vehicle Synthetic Aperture Radar (UAVSAR) is an airborne L-band polarimetric radar system developed at the Jet Propulsion Laboratory [40]. L-band (23 cm wavelength) radars have greater penetrating capabilities and sensitivity to tree trunks than smaller wavelengths. Fully polarimetric capabilities i.e., the ability to record four combinations of transmitted and received polarized signals make this data set particularly useful for studying surface and volume scattering from vegetation as well as structural properties such as Leaf Area Index (LAI), basal area and biomass [41]–[43]. While many studies have suggested the use of polarimetric L-band radar data by itself and in combination with lidar for habitat mapping, few studies have actually tested them. Here, we used co-polarized [horizontal transmitted, horizontal received HH], [vertical transmitted, vertical received VV] and cross-polarized [horizontal transmitted, vertical received HV] data. Raw data were processed into backscatter images at 5 m nominal spatial resolution, orthorectified with digital elevation models and corrected for topographic slope (i.e. area projection in the line of sight). We converted backscatter values into power, applied a (3×3) gamma filter to reduce fine-scale variations, and calculated average statistics for HH, VV, and HV bands within bird plots [44]. In addition, band ratios HH/VV, HV/VV HV/HH and normalized difference band ratios [HH-VV/HH+VV], [VV-HV/VV+HV], and [HH-HV/HH+HV] were calculated [45].

All datasets were brought into a common frame of reference using the UTM 19 N projection and WGS 84 datum. Landsat ETM+ data and UAVSAR were geo-referenced using DEMs while DRL and LVIS data were geolocated using GPS and inertial navigation units. The geolocation error of each dataset was evaluated individually and found to be less than 1 pixel i.e. Landsat ETM+ was less than 30 m, DRL data was within 0.5 m, LVIS was less than 25 m and UAVSAR was less than 5 m. While shifts between datasets of varying resolutions were unavoidable, they were well within the bird plot scale of 0.79 ha. Averaging remote sensing data attributes over 88.8 m pixels (0.79 ha area) further minimized errors. One bird plot was excluded because of no co-incident LVIS data and another because of high DRL crown delineation error leaving 369 bird plots and 104 predictor variables (Table 2) for analyses. Prevalence was calculated as the total number of years a bird was detected in a plot out of the 9 years observed, i.e., the lowest prevalence was zero and maximum was 9. Prevalence was predicted as a continuous variable following [1].

**Table 2:** Predictor variables calculated from the different remote sensing data sets.

Metrics used	(Min.,Max.,Mean.,Std.deviation)
Radar	HV, HH, HV backscatter
	HH/VV, HV/VV, HV/HH ratios
	[HH-VV/HH+VV] index
	[HH-HV/HH+HV] index
	[VV-HV/VV+HV] index
Landsat	NDVI (only Mean,Std.deviation)
	NDVI change (only Mean,Std.deviation)
LVIS	Elevation (only Mean,Std.deviation)
	RH metrics (RH25, RH50,RH75,RH100)
	Total Canopy Cover
	Canopy cover at 5 m intervals from 0 to 35 m
DRL	Height of individual trees identified with TreeVaW
	Crown diameters of individual trees
	Stem density (stems/ha)
	Crown area weighted height
	Product of height and crown diameter

### Analysis

Machine learning algorithms such as decision trees and neural networks do not make any assumptions about the relationships between explanatory and response variables and are well suited for analyzing complex non-linear and possibly hierarchical interactions in large ecological data sets [46]. Decision/regression trees [47] for example, partition the data into two homogenous sets based on the best explanatory variable. The binary tree is further subdivided recursively using decision rules until a terminal node is reached, providing a mean value for the response variable. Random Forests, RF [48] introduces randomness in the selection of the best split by testing a random subset of predictors at each node, generally 1/3 the total number of variables [49]. A large number of such regression trees are constructed using bootstrap samples of the dataset for each tree and random subsets of variables at each node, hence the name “Random Forests.” The remaining 37% of the data after bootstrapping form the “out-of-bag” (OOB) observations. These observations are run through each regression tree to predict responses and calculate out-of-bag error estimates [48], [50]. Because OOB observations are not used to construct trees, they essentially provide cross-validated errors and by averaging errors over hundreds of trees, the possibility of over-fitting is considerably reduced. Predictions from RF models are often more accurate than other methods and are increasingly used in ecological applications such as modeling species distributions [51], [52] and bird habitat quality [1]. We constructed 5 RF models (Random Forests R package) for each bird species with predictor variables from radar, Landsat, DRL, LVIS, and fusion resulting in a total of 40 models predicting prevalence for all species. We compared the decrease in mean residual error with increasing number of trees (100 to 8,000) and found that 800 trees gave the best predictions. Growing more than 800 trees did not improve predictive power for any species. The number of variables sampled at each node split was set to the default of one-third the total variables in each model as changing this parameter did not significantly improve the variance explained by the model. Prediction accuracies were assessed using percent variance in bird prevalence explained by each model. Variance explained, also known as pseudo-r^2^ was calculated as 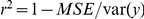 where *MSE* is the mean square error between observed values (y) and out-of-bag predictions [48], [53]. In addition to the pseudo-r^2^, variable importance was calculated from RF models as the increase in MSE error on removing a predictor variable from out of bag predictions. If the increase in error on removing a variable was large, it indicated high importance and vice versa [48]. The frequency of occurrence of a radar, lidar, or Landsat metric within the 10 most important variables in each fusion model was recorded to determine which variables were more useful in predictions. We then predicted prevalence across the landscape for the 8 bird species and compared spatial variations with known bird habitat preferences.

## Results

**Figure 2:**
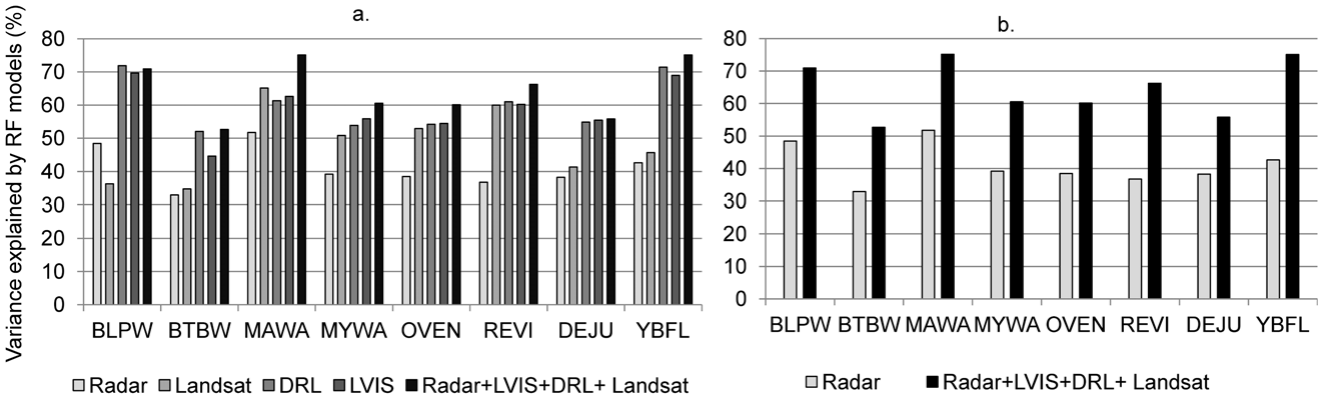
Random Forests accuracies for all models (a). Comparison of lowest accuracy [radar] and highest accuracy [fusion] models (b).

**Figure 3:**
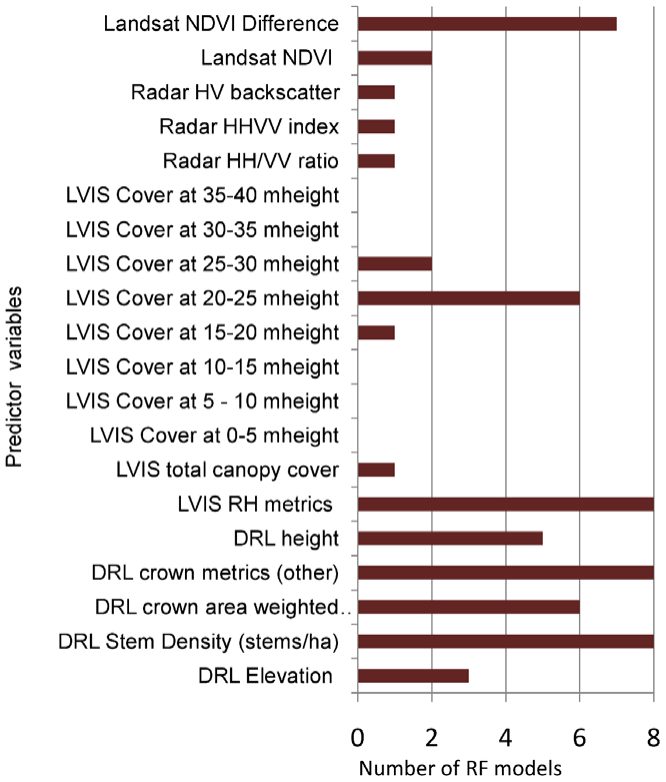
Predicted prevalence for all species from fusion. Combined pseudo-r^2^ calculated from out of bag errors = 77%.

Radar metrics alone explained more than 30% variance in prevalence for all species, with higher accuracies for magnolia warbler (50%) and blackpoll warbler (48%) (Fig. 2a). Landsat metrics outperformed radar (35% to 65%) and were better than lidar in the case of the magnolia warbler. Lidar metrics from LVIS and DRL predicted prevalence with more than 50% accuracy for all species (54%–71%), except for the black-throated blue warbler. Fusion explained between 52% and 75% variance in prevalence and improved the predictive power of radar by 25% (Fig. 2b), Landsat by 15%, and lidar by 4% on an average. A combined pseudo- r2 was calculated using the prediction errors (MSE) from all fusion models. Results showed that fusion explained more than 77% variance in the combined prevalence for all the birds (Fig. 3).

**Figure 4:**
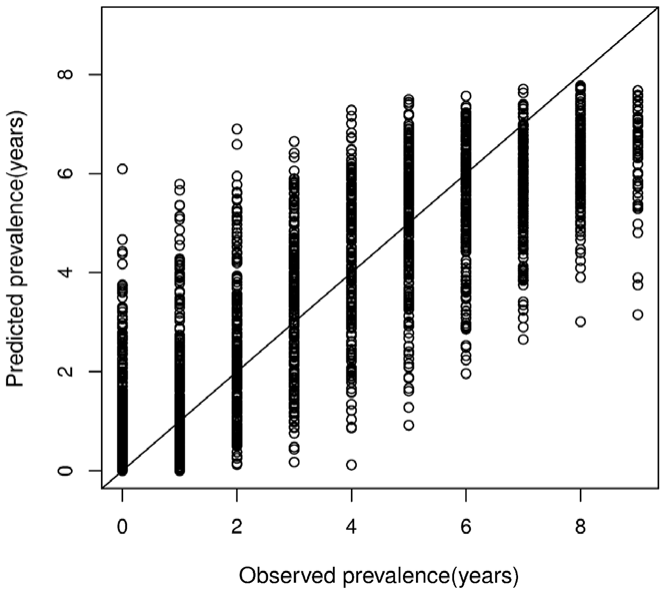
Frequency of variable selection within the 10 most important predictors for each bird species. Model used: [Radar+Landsat+DRL+LVIS].

**Figure 5:**
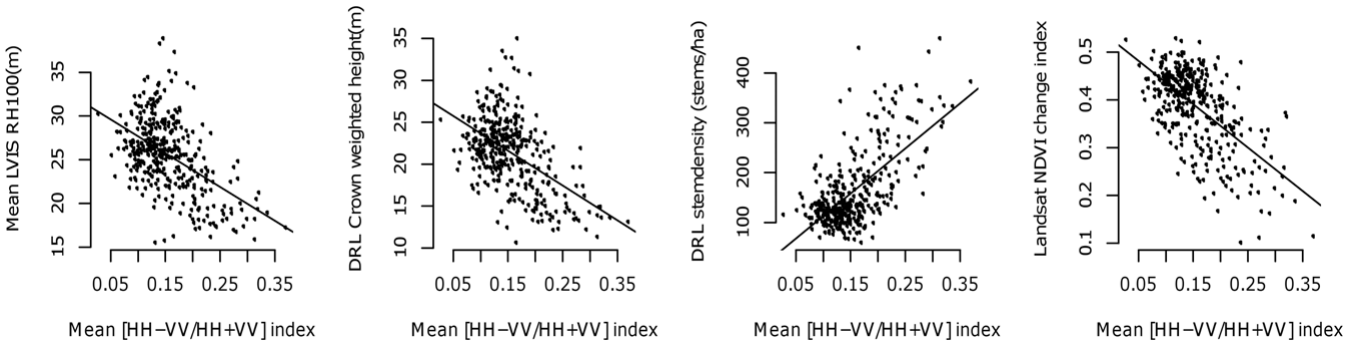
Correlation between radar backscatter ratios and other remote sensing variables. Significant at p = 0.05.

Next, we analyzed the frequency of variable selection among the 10 most important predictors for each species (Fig. 4). Stem density and crown metrics derived from DRL were selected as important variables in all models. The NDVI change or deciduousness metric from Landsat was the best predictor for the magnolia warbler and important for 7 out of 8 species. Radar metrics were rarely selected when used in combination with other datasets. When selected, co-polarized backscatter ratios, particularly the HH/VV ratio, the (HH-VV/HH+VV) index and the HV/VV metric were more important than other radar metrics. These indices were also correlated with LVIS heights and crown characteristics from DRL (Fig. 5). LVIS relative height (RH) metrics were useful predictors in all models. RH75 in particular was strongly related to DRL crown weighted height.

**Figure 6:**
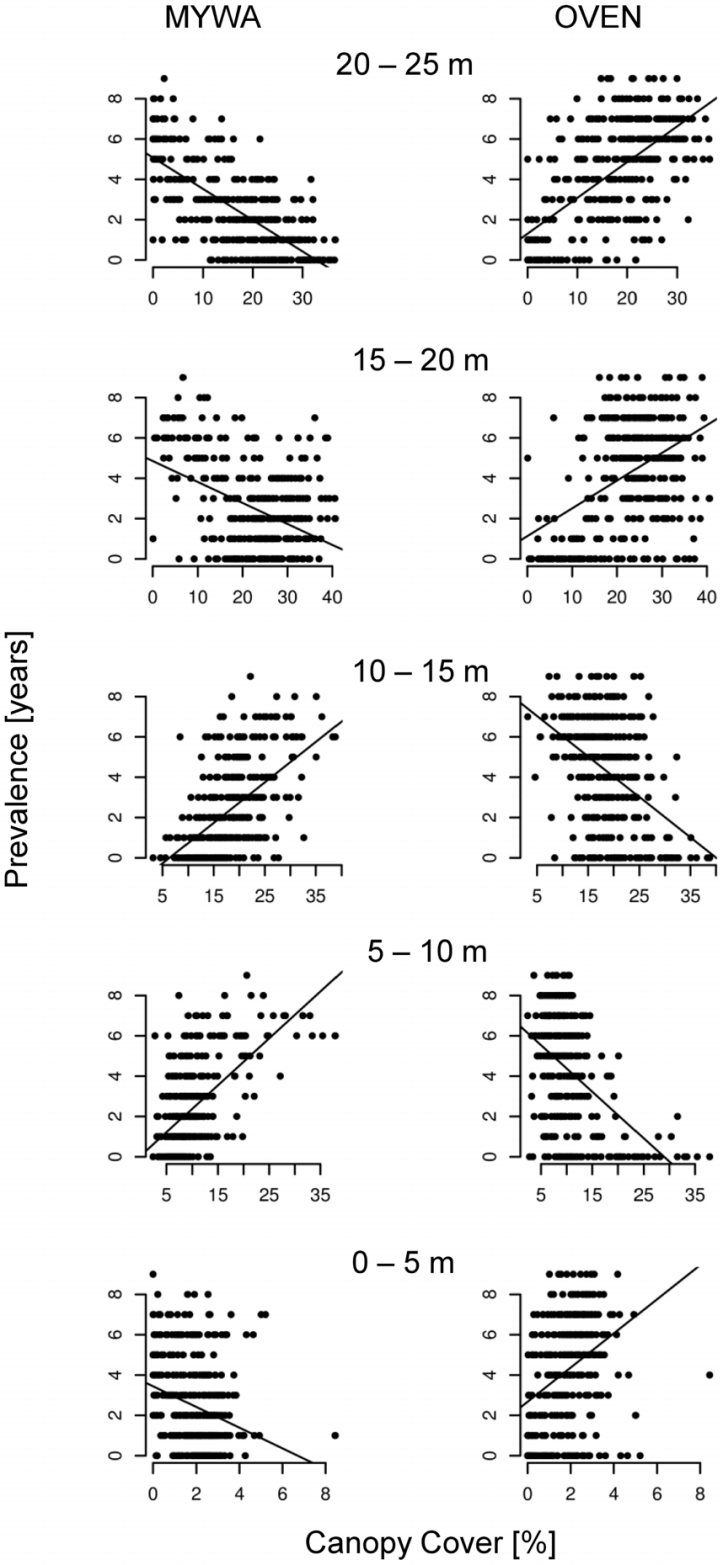
Variations in prevalence with canopy cover at different height intervals for the yellow rumped warbler [MYWA] and ovenbird [OVEN]. Regression lines significant at p = 0.05. Note variable scaling of x-axis.

Contrary to our expectations, canopy cover metrics from LVIS waveforms were not as useful as RH metrics in predicting prevalence. We did, however, note variations in prevalence with canopy cover (Fig. 6). For example, prevalence in the yellow rumped warblers was positively correlated with canopy cover between 5–10 m and 10–15 m and negatively correlated below 5 m and above 15 m. Similar variations were observed for blackpoll warbler, magnolia warbler, dark- eyed junco and the yellow-bellied flycatcher. The ovenbird, on the other hand, showed negative correlation with canopy cover between 5–10 m and 10–15 m and positive correlation below 5 m and above 15 m. The red-eyed vireo and black-throated blue warbler (BTBW) showed similar variations with canopy cover, although relationships were much weaker in the case of the BTBW. The correlation between prevalence and canopy cover was significant (p = 0.05) at all height intervals.

**Figure 7:**
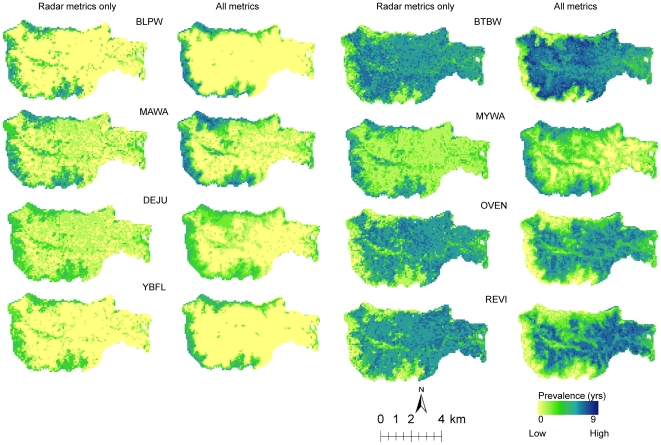
Spatial predictions of bird prevalence from models with lowest [radar] and highest accuracies [fusion]. Refer to table 1 for species codes.

Maps of prevalence (Fig. 7) at the landscape level showed that predictions from both radar alone as well as from fusion were consistent with known habitat characteristics [29], [54] and showed three general spatial patterns. The blackpoll warbler, magnolia warbler, dark- eyed junco, and yellow-bellied flycatcher showed highest prevalence around the perimeter of the Hubbard Brook valley, corresponding to higher elevation coniferous forests with high stem density and low canopy heights. The black-throated blue warbler, red-eyed vireo, and ovenbird occurred more in the central portions of the valley, characterized by deciduous forests with tall trees, dense overstory canopy cover, and lower stem density. A third intermediate and patchy pattern was noted in the case of the yellow rumped warbler (Fig. 7). Other species not considered here also show this broader pattern [29]. While spatial patterns of low and high prevalence were similar from radar and fusion (Fig. 7), there were considerable variations within each prevalence class.

**Figure 8:**
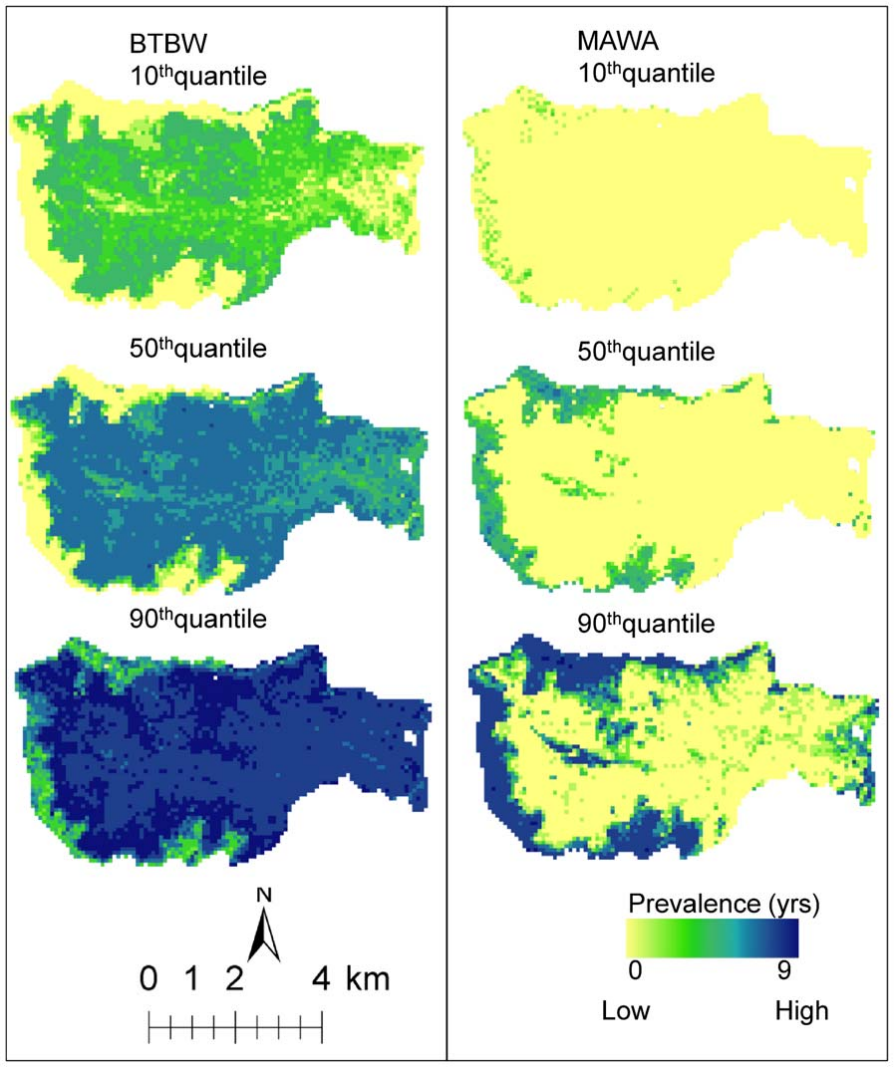
Quantile predictions for black-throated blue warbler [BTBW] and magnolia warbler [MAWA].

Lastly, we examined the uncertainty in RF models using quantile regression. Although RF models can have high prediction accuracies, they retain only the mean response for each observation and these responses per se may not be sufficient for ecological interpretation. Quantile regression is useful for analyzing model uncertainties, detecting outliers, and exploring causal relationships that may not be detected in mean responses alone [55]. Quantile regression forests (QRF) is a generalization of RF, where all observations are saved to provide a non-parametric distribution of predicted values [56], and quantiles from this distribution can be used to construct prediction intervals to assess model spatial uncertainties. We predicted the 10^th^, 50^th^, and 90^th^ quantiles for the black throated blue warbler and magnolia warbler using QRF (Quantile Regression Forests R Package) and mapped the distributions over the HBEF (Fig. 8). Results showed a wide range of predictions for BTBW prevalence. The 10^th^ quantile showed more predictions of low and medium prevalence but very few areas with high prevalence. On the other hand, the 90^th^ quantile showed high prevalence over the entire study area with only a few patches of medium prevalence along the perimeter. The 50^th^ quantile map closely matched the mean predictions from RF regression (Fig. 7 and Fig. 8). In the case of the magnolia warbler, the 10^th^ quantile showed little or no prevalence while the 90^th^ quantile showed high prevalence along the perimeter of the study area.

## Discussion

Our primary objective was to compare the efficacies of different remote sensing data sets in mapping bird prevalence across a forested landscape. Multi-sensor fusion explained variations in prevalence with higher accuracies than from any one sensor alone because each data set provided some complementary attributes not available in the others. The pseudo-r^2^ (77%) for all species together as an ensemble was higher than that for any one individual species (52%–74%). This can occur because the ensemble predictions cover the entire range of prevalence values, increasing the overall variance explained. The predictive capability of each sensor varied based on its sensitivity to vegetation spatial and vertical structure as well as composition (which may be indicative of habitat characteristics affecting prevalence).

RF models developed from radar data alone showed the lowest prediction accuracies for all birds because radar backscatter ratios were only moderately correlated with structural and compositional metrics (inferred from the other sensors) (Fig. 5). Backscatter is affected by several factors including steep terrain, variable soil moisture, and high canopy density all of which were present at HBEF and may have reduced the predictive capability of radar. Aboveground biomass is an important indicator of age, successional status, and productivity of an ecosystem [9]. Average aboveground biomass values in the HBEF are around 203.5 Mg/ha and as low as 50 Mg/ha at higher elevations [57]. Radar backscatter is sensitive to canopy structure for low biomass forests but saturates in high biomass areas. This could possibly explain the higher power of radar variables in explaining bird prevalence in low biomass forests (e.g. blackpoll warbler, magnolia warbler and yellow- bellied flycatcher) as compared to those in mature, high biomass forests (e.g. black-throated blue warbler, ovenbird, and red-eyed vireo).

Multispectral Landsat data outperformed radar data and were comparable to accuracies from lidar for 4 out of the 8 species studied. This was most likely because of the seasonal NDVI change metric that accounted for the steep conifer-deciduous gradient in the study area. This implies that deciduousness was as important as structure for the bird species considered. However, deciduousness is reflective of differences between coniferous and deciduous forests that include plant species composition, abundance of associated arthropod fauna (important as food for birds), canopy structure, and other edaphic and climatic factors. We cannot determine through our analyses here which of these were important differentiators of habitat preference.

We know that lidar metrics predicted prevalence with higher accuracies than radar and Landsat for most species, which implies that canopy height, canopy cover, and crown characteristics are key habitat metrics for these species. There was little difference between results from LVIS and DRL suggesting that large footprint waveform data could be just as effective as high-resolution discrete return lidar in explaining variations in habitats. This is somewhat counter-intuitive; we expected that highly detailed canopy structure information at the scale of individual trees and gaps would have better predictive power than the LVIS data. In one sense, this expectation was met: fewer DRL metrics explained the same variations in prevalence as a large number of metrics from waveform lidar suggesting an advantage of high-resolution DRL data in habitat mapping. Note, however, that waveforms from an instrument such as LVIS are generated from photon interactions that occur at the finest scales and are aggregated at any height across a footprint. Thus, waveform metrics are correlated with stand level canopy measures, such as tree density, that are derived from canopy structures much finer than the footprint resolution. For example, RH75 can be correlated to basal-area weighted height (Lorey's height) at the plot level. So even though LVIS does not measure the number of trees directly, which DRL can do, the waveform implicitly captures this information. This has important implications for habitat mapping from space-borne sensors with footprint sizes far exceeding DRL. In any case, the question of the appropriate scale (grain) of canopy measurement for habitat analyses is still an open one that will require more study.

Our analyses using RF allowed us to assess the importance of the individual remote sensing variables and thus habitat characteristics in predicting prevalence. Out of 104 variables, stem density and crown metrics from DRL data, seasonal NDVI change from Landsat and LVIS quantile energy (RH) metrics were selected more often than other variables. A previous study [3] found that deciduousness from Landsat, canopy height metrics from LVIS and elevation were important predictors of prevalence for one species (the black-throated blue warbler) [1]. Our work is consistent with this result and shows that these metrics were useful for the other bird species as well. Identifying the most important structural and compositional variables influencing habitat use as demonstrated here provides a quantitative basis for identifying and protecting existing high use sites as well as for managing habitats for multiple species, especially under competing policy scenarios. These important variables may further be used in a hierarchical multi-species model to predict occurrence for species assemblages, as shown by [58].

The availability of multi-dimensional habitat characteristics from remote sensing at landscape scales can provide new perspectives on habitat data. A novel finding in our study was the variation in bird prevalence with canopy cover at 5 m height intervals. Ovenbird and yellow-rumped warbler prevalence showed exactly opposite relationships with canopy cover at each interval suggesting preferences for dissimilar habitats. Variations in red-eyed vireo and black-throated blue warbler prevalence were similar to the ovenbird while those of the blackpoll warbler, magnolia warbler, dark-eyed junco, and yellow-bellied flycatcher were similar to the yellow- rumped warbler. While these species are known to co-exist at the HBEF, our study supports a long-standing hypothesized mechanism for coexistence, i.e. habitat preferences based on vertical variations in cover [4].

Within the same species, birds showed opposite preferences for cover between 5–15 m and 15–25 m indicating stratification based on foliage density. Previous field-based studies [6] have divided the canopy in the HBEF into three strata: canopy (15–27 m), sub-canopy (2–14 m), and shrub (<2 m), based on vertical foliage distributions. Foliage in the canopy stratum is relatively dense while the sub-canopy stratum is open and sparse. The positive correlation of ovenbird prevalence with cover in the 15–25 m stratum suggests preference for dense overstory while negative correlation between 5–15 m indicates preference for open sub-canopy. The yellow-rumped warbler, on the other hand, was more prevalent in dense sub-canopy and open overstory. Changes in the relative densities of foliage in these layers during forest succession have been proposed to be a major factor affecting the abundance of bird species in the HBEF [24]. Our results demonstrate these relationships can be quantified with lidar data at fine vertical and spatial scales. Such information could be exceptionally useful in understanding how forest succession and resulting changes in foliage densities affect bird populations.

The patchiness and spatial variations in prevalence were similar to patterns of bird abundance from a previous field-based study [29] showing spatial heterogeneity. The distinct division of birds into groups was noted both vertically, based on canopy cover preferences and spatially based on deciduousness, stem density and other habitat preferences. These results demonstrate the usefulness of remote sensing in quantifying habitat heterogeneity as well as mapping bird guilds.

According to [29], bird abundance patterns were spatially auto-correlated for several species in the HBEF. We did not explicitly model spatial autocorrelation in this study because the focus of our study was an inter-sensor comparison. Analyses of correlograms from RF model residuals showed markedly reduced autocorrelation (from about 0.7 to 0.2 at the shortest lags) similar to [59]. However, Moran's I co-efficients though small (and generally monotonically decreasing) were statistically significant (p = 0.05) at more than one lag distance suggesting that RF models may not entirely account for spatial autocorrelation.

Characterizing the confidence of spatial predictions from machine learning methods such as RF is a critical task, though ignored in many studies. The quantile maps shown (Fig. 8) provide a prediction interval that quantifies this model-based uncertainty across the landscape. The 10th quantile gives a low (conservative) estimate of prevalence (i.e. we see a lower prevalence value only 10% of the time). Thus, areas that score highly on the 10% map are probably very good sites with regard to prevalence. Conversely, the 90th quantile gives an optimistic view of prevalence; if an area scores low on this map it is likely that this is a poor site with respect to prevalence. For example, there is a wide range of predictions for the black-throated blue warbler over much of the HBEF indicating somewhat weak mean predictions [55], [56] (specific locations could be classified anywhere between low and high prevalence). The magnolia warbler prediction intervals, in contrast, are narrower suggesting stronger mean predictions. The information available from quantile maps also has implications for management. If limited management resources are available for habitat preservation and protection, the case could be made that these should be directed towards areas of high prevalence where model predictions are most confident.

Maps produced solely from radar broadly identified general patterns of prevalence but with lower accuracy as compared to maps produced from fusion, which had higher accuracies and captured subtle variations that were not detected by radar alone. For example, radar maps showed little difference between blackpoll warbler, dark eyed junco, magnolia warbler, and yellow-bellied flycatcher prevalence (Fig. 7), whereas those from fusion identified finer scale variations based on deciduousness, and other forest structural characteristics. Predictions from radar metrics were also similar for black-throated blue warbler, red-eyed vireo, and ovenbird, but maps from fusion showed that habitats with high black-throated blue warbler prevalence were clearly different from those for red-eyed vireo and ovenbird. These results suggest that radar data alone can be used to identify broad scale habitat characteristics but finer differentiation likely requires other types of remote sensing data, such as lidar.

Our results demonstrate the potential of multi-sensor fusion in comprehensive habitat monitoring for wildlife species. Most habitat studies are limited to fine scale field-based studies at stand level and coarse predictions at landscape level. Lidar substantially reduces the typical trade-off between spatial resolution and extent of vegetation data in species distribution studies by providing high-resolution habitat characteristics at both scales. While wall-to-wall coverage of lidar data is preferred for ecological applications, in practice, both waveform and discrete return lidar are sparsely available. Radar data with larger cloud free coverage and sensitivity to vegetation structure is an attractive alternative but our results show that radar by itself may not be sufficiently effective. Multispectral data and spatially sparse lidar samples might be used to calibrate radar data to extensively map structural characteristics [9]. The density of lidar samples required for driving radar-based models still needs to be explored. In the absence of lidar data, simple backscatter ratios from radar may be also useful for rapid habitat assessment with lower accuracies.

Lastly, this study highlights the enormous potential of multi-sensor fusion in advancing field- based ecological and habitat studies. The multi-dimensional habitat characteristics generated from lidar and fusion powerfully augment field-based studies and can help ecologists explore questions on species-habitat relationships in new and previously unexplored ways.

## References

[1] Goetz SJ, Daniel S, Betts MG, Holmes RT, Doran PJ (2010). Lidar remote sensing variables predict breeding habitat of a Neotropical migrant bird.. Ecology.

[2] Caillaud D, Crofoot MC, Scarpino SV, Jansen PA, Garzon-Lopez CX (2010). Modeling the Spatial Distribution and Fruiting Pattern of a Key Tree Species in a Neotropical Forest: Methodology and Potential Applications.. PLoS ONE.

[3] Betts MG, Rodenhouse NL, Scott Sillett T, Doran PJ, Holmes RT (2008). Dynamic occupancy models reveal within-breeding season movement up a habitat quality gradient by a migratory songbird.. Ecography.

[4] MacArthur R, MacArthur J (1961). On bird diversity.. Ecology.

[5] Degraaf R (1998). Associations between breeding bird abundance and stand structure in the White Mountains, New Hampshire and Maine, USA.. Forest Ecology and Management.

[6] Robinson SK, Holmes RT (1984). Effects of Plant Species and Foliage Structure on the Foraging Behavior of Forest Birds.. Auk.

[7] Doran PJ, Holmes RT (2005). Habitat occupancy patterns of a forest dwelling songbird: causes and consequences.. Canadian Journal of Zoology.

[8] Bradbury R, Hill R, Mason D, Hinsley S, Wilson J (2005). Modelling relationships between birds and vegetation structure using airborne LiDAR data: a review with case studies from agricultural and woodland environments.. Ibis.

[9] Bergen KM, Goetz SJ, Dubayah RO, Henebry GM, Hunsaker CT (2009). Remote sensing of vegetation 3-D structure for biodiversity and habitat: Review and implications for lidar and radar spaceborne missions.. Journal of Geophysical Research.

[10] Hall DL, Llinas J (1997). An introduction to multisensor data fusion.. Proceedings of the IEEE.

[11] Pohl C, Van Genderen JL (1998). Review article Multisensor image fusion in remote sensing: concepts, methods and applications.. International Journal of Remote Sensing.

[12] Mcdermid GJ, Franklin SE, Ledrew EF (2005). Remote sensing for large-area habitat mapping.. Progress in Physical Geography.

[13] Moody A, Johnson DM (2001). Land-surface phenologies from AVHRR using the discrete fourier transform.. Remote Sensing of Environment.

[14] Gustafson EJ (1998). Quantifying landscape spatial pattern: What is the state of the art?. Ecosystems.

[15] Lefsky MA, Cohen WB, Parker GG, Harding DJ (2002). Lidar Remote Sensing for Ecosystem Studies.. BioScience.

[16] Hyde P, Dubayah R, Walker W, Blair JB, Hofton M (2006). Mapping forest structure for wildlife habitat analysis using multi-sensor (LiDAR, SAR/InSAR, ETM+, Quickbird) synergy.. Remote Sensing of Environment.

[17] Goetz S, Steinberg D, Dubayah R, Blair B (2007). Laser remote sensing of canopy habitat heterogeneity as a predictor of bird species richness in an eastern temperate forest, USA.. Remote Sensing of Environment.

[18] Vierling K, Vierling L, Gould W, Martinuzzi S, Clawges R (2008). Lidar: shedding new light on habitat characterization and modeling.. Frontiers in Ecology and the Environment.

[19] Dubayah RO, Drake JB (2000). Lidar remote sensing for forestry applications.. Journal of Forestry.

[20] Bergen KM, Gilboy AM, Brown DG (2007). Multi-dimensional vegetation structure in modeling avian habitat.. Ecological Informatics.

[21] Imhoff M (1997). Remotely sensed indicators of habitat heterogeneity: Use of synthetic aperture radar in mapping vegetation structure and bird habitat.. Remote Sensing of Environment.

[22] Holmes RT, Bonney REJ, Pacala SW (1979). Guild Structure of the Hubbard Brook Bird Community: A Multivariate Approach.. Ecology.

[23] Whelan CJ (2001). Foliage Structure Influences Foraging of Insectivorous Forest Birds: An Experimental Study.. Ecology.

[24] Holmes RT, Sherry TW (2001). Thirty-Year Bird Population Trends in an Unfragmented Temperate Deciduous Forest: Importance of Habitat Change.. The Auk.

[25] Holmes RT (2010). Avian population and community processes in forest ecosystems: Long-term research in the Hubbard Brook Experimental Forest.. Forest Ecology and Management.

[26] Schwarz P (2001). Structure and composition of three northern hardwood–conifer forests with differing disturbance histories.. Forest Ecology and Management.

[27] Schwarz PA, Fahey TJ, Mcculloch CE (2003). Factors Controlling Spatial Variation of Tree Species Abundance in a Forested Landscape.. Ecology.

[28] Ralph JC, Sauer JR, Droege S (1995). Monitoring Bird Populations by Point Counts..

[29] Doran PJ (2003). Intraspecific spatial variation in bird abundance: patterns and processes..

[30] Goetz SJ (1997). Multi-sensor analysis of NDVI , surface temperature and biophysical variables at a mixed grassland site.. International Journal of Remote Sensing.

[31] Drake J, Dubayah R, Clark DB, Knox RG, Blair JB (2002). Estimation of tropical forest structural characteristics using large-footprint lidar.. Remote Sensing of Environment.

[32] Clark ML, Clark DB, Roberts DA (2004). Small-footprint lidar estimation of sub-canopy elevation and tree height in a tropical rain forest landscape.. Remote Sensing of Environment.

[33] Popescu SC, Wynne RH, Nelson RF (2003). Measuring individual tree crown diameter with LiDAR and assessing its influence on estimating forest volume and biomass.. Canadian Journal of Remote Sensing.

[34] Anderson J, Martin ME, Smith M-L, Dubayah RO, Hofton MA (2006). The use of waveform lidar to measure northern temperate mixed conifer and deciduous forest structure in New Hampshire.. Remote Sensing of Environment.

[35] Popescu SC, Wynne RH, Scrivani JA (2004). Fusion of Small-Footprint Lidar and Multispectral Data to Estimate Plot- Level Volume and Biomass in Deciduous and Pine Forests in Virginia, USA.. Biomass.

[36] Pang Y, Lefsky M, Andersen H-E, Miller ME, Sherrill K (2008). Validation of the ICEsat vegetation product using crown-area-weighted mean height derived using crown delineation with discrete return lidar data..

[37] Blair JB, Rabine DL, Hofton MA (1999). The Laser Vegetation Imaging Sensor: a medium-altitude, digitisation-only, airborne laser altimeter for mapping vegetation and topography.. ISPRS Journal of Photogrammetry and Remote Sensing.

[38] Dubayah RO, Sheldon SL, Clark DB, Hofton MA, Blair JB (2010). Estimation of tropical forest height and biomass dynamics using lidar remote sensing at La Selva, Costa Rica.. J Geophys Res.

[39] Ni-Meister W, Jupp DLB, Dubayah R (2001). Modeling lidar waveforms in heterogeneous and discrete canopies.. IEEE Transactions on Geoscience and Remote Sensing.

[40] Rosen PA, Hensley S, Wheeler K, Sadowy G, Miller T (2006). UAVSAR: A New NASA Airborne SAR System for Science and Technology Research.. IEEE Conference on Radar.

[41] Antonarakis AS, Saatchi SS, Chazdon RL, Moorcroft PR (in press). Using Lidar and Radar measurements to constrain predictions of forest ecosystem structure and function.. Ecological Applications.

[42] Moghaddam M, Saatchi S (2000). Estimation of crown and stem water content and biomass of boreal forest using polarimetric SAR imagery.. IEEE Transactions on Geoscience and Remote Sensing.

[43] Treuhaft RN, Law BE, Asner GP (2004). Forest Attributes from Radar Interferometric Structure and Its Fusion with Optical Remote Sensing.. BioScience.

[44] Goncalves FG, Santos JR, Treuhaft RN (2011). Stem volume of tropical forests from polarimetric radar.. International Journal of Remote Sensing.

[45] Simental E (2005). Polarimetry Band Ratios, Decompositions and Statistics for Terrain Characterization..

[46] Olden JD, Lawler JJ, Poff NL (2008). Machine learning methods without tears: a primer for ecologists.. The Quarterly review of biology.

[47] De'ath G, Fabricius KE (2000). Classification and Regression Trees: A Powerful Yet Simple Technique for Ecological Data Analysis.. Ecology.

[48] Breiman L (2001). Random Forests.. Machine Learning.

[49] Prasad AM, Iverson LR, Liaw A (2006). Newer Classification and Regression Tree Techniques: Bagging and Random Forests for Ecological Prediction.. Ecosystems.

[50] Berk R (2008). An Introduction to Statistical Learning from a Regression Perspective.. Analysis.

[51] Magness DR, Huettmann F, Morton JM (2008). Using Random Forests to Provide Predicted Species Distribution Maps as a Metric for Ecological Inventory & Monitoring Programs.. Studies in Computational Intelligence (SCI).

[52] Cutler DR, Edwards TC, Beard KH, Cutler A, Hess KT (2007). Random forests for classification in ecology.. Ecology.

[53] Wei C-L, Rowe TG, Escobar-Briones E, Boetius A, Soltwedel T (2010). Global Patterns and Predictions of Seafloor Biomass Using Random Forests.. PLoS ONE.

[54] Betts M, Diamond A, Forbes G, Villard M, Gunn J (2006). The importance of spatial autocorrelation, extent and resolution in predicting forest bird occurrence.. Ecological Modelling.

[55] Cade BS (2003). A Gentle Introduction to Quantile Regression for Ecologists.. Frontiers in Ecology and the Environment.

[56] Meinshausen N (2006). Quantile Regression Forests.. Journal of Machine Learning Research.

[57] Fahey TJ, Siccama TG, Driscoll CT, Likens GE (2005). The biogeochemistry of carbon at Hubbard Brook.. Biogeochemistry.

[58] Zipkin E, Andrew R, Dawson D, Scott B (2010). Multi-species occurrence models to evaluate the effects of conservation and management actions.. Biological Conservation.

[59] Cablk ME, White D, Kiester AR, Scott M, Heglund P, Morrison M, Rafael M, Wall B, Hoffer J (2002). Assessment of spatial autocorrelation in empirical models in ecology..

